# Engineered Mesenchymal Stem Cell–NK Cell Complexes for Spatially Targeted and Functionally Revitalized Cancer Immunotherapy

**DOI:** 10.1002/advs.202509638

**Published:** 2025-08-19

**Authors:** Qian Zhang, Bo Yin, Munaiwaier Sabier, Youlan Yang, Mengling Wu, Zhouping Zhao, Xuanzhi Luo, Yuehong Zhong, Xiuming Zhu, Jie Zhang, Jing Wang, Kai Chen, Fei Ruan, Wei Zhang, Zhimin Lu, Jiong Wang

**Affiliations:** ^1^ Department of Geriatric Respiratory and Critical Care The First Affiliated Hospital of Anhui Medical University Hefei 230022 China; ^2^ Department of General Practice The First Affiliated Hospital of Anhui Medical University Hefei 230022 China; ^3^ Department of Geriatrics Renhe Hospital of Shanghai University Shanghai 200431 China; ^4^ Department of Geriatrics Center & National Clinical Research Center for Aging and Medicine Jing'an District Central Hospital of Shanghai Fudan University Shanghai 200040 China; ^5^ Department of Obstetrics and Gynecology the First Affiliated Hospital of Anhui Medical University Hefei 230022 China; ^6^ Institute of Medical Genetics and Development Key Laboratory of Reproductive Genetics (Ministry of Education) and Women's Hospital Zhejiang University School of Medicine Hangzhou 310006 China; ^7^ Department of Gynecology Women's Hospital Zhejiang University School of Medicine Hangzhou 310006 China

**Keywords:** bioorthogonal click chemistry, Galectin‐9 blockade, mRNA transfection, mesenchymal stem cells, NK cell therapy

## Abstract

Natural killer (NK) cells represent a powerful immunotherapeutic strategy due to their intrinsic cytotoxicity and ability to target tumor cells independently of antigen presentation. However, their clinical efficacy against solid tumors is limited by poor tumor infiltration and impaired functionality within the immunosuppressive microenvironment. Here, a genetically engineered cell‐cell complex delivery system comprising NK cells conjugated to IL‐15 expressing adipose‐derived mesenchymal stem cells (SCs) is developed. By exploiting SCs’ intrinsic tumor‐tropic properties and engineering them to consistently express interleukin‐15 (IL‐15) via lipid nanoparticle‐mediated mRNA transfection, the SC‐NK cell complexes exhibit markedly improved tumor localization and sustained cytokine‐mediated functional enhancement. Utilizing bioorthogonal click chemistry for precise conjugation, the approach effectively enhances NK cell infiltration and revitalizes their cytotoxic activity in both orthotopic murine lung cancer and patient‐derived xenograft ovarian cancer models. Furthermore, increased responsiveness of the cell‐cell complexes to Galectin‐9 blockade therapy is identified, leading to the reversal of NK cell dysfunction and significantly augmented antitumor efficacy. Collectively, the engineered SC‐assisted delivery system holds potential for overcoming the limitations of NK cell therapies and improving their therapeutic outcomes in solid tumor.

## Graphical abstract

IL‐15‐engineered stem cell–NK cell complexes, assembled via bioorthogonal chemistry, enable effective lung cancer immunotherapy.

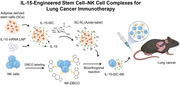

## 1 Introduction

Natural killer (NK) cells have emerged as a promising strategy against malignancies, demonstrating remarkable success in both preclinical and clinical settings.^[^ ^1^ ^]^ Unlike T cells, NK cells can recognize and eliminate malignant cells in an antigen‐independent manner, a feature that enables them to target tumor cells that have downregulated major histocompatibility complex class I (MHC‐I) molecules—a common immune evasion strategy employed by cancers.^[^ ^2^, ^3^ ^]^ This intrinsic cytotoxicity, combined with their ability to respond rapidly without the need for prior sensitization, underscores one of the greatest advantages of NK cell‐based therapies.^[^ ^4^ ^]^ Despite these advantages, their application in solid tumors has been hampered by several intrinsic and extrinsic barriers. NK cells often exhibit poor infiltration into tumor sites, largely due to mismatched chemokine receptor profiles (e.g., low CXCR3/CXCR4 expression) that fail to respond to tumor‐derived chemotactic signals.^[^ ^5^, ^6^ ^]^ Once within the tumor microenvironment (TME), NK cells undergo functional exhaustion caused by inhibitory receptor engagement (e.g., NKG2A‐HLA‐E interactions), metabolic competition (e.g., adenosine accumulation), and exposure to immunosuppressive cytokines (e.g., TGF‐*β* and IL‐10).^[^ ^7^, ^8^, ^9^ ^]^ Additionally, Galectin‐9 (Gal‐9), a *β*‐galactoside‐binding lectin overexpressed in various tumors, modulates the immune microenvironment by interacting with TIM‐3 receptors on NK cells.^[^ ^10^, ^11^ ^]^ This inhibitory interaction triggers signaling pathways that exacerbate NK cell dysfunction and promote immune evasion by inducing apoptosis and suppressing cytotoxic activity.^[^ ^12^ ^]^ These converging mechanisms collectively impair NK cell cytotoxicity, proliferation, and persistence, highlighting the urgent need for innovative strategies to enhance both the targeted delivery and functional resilience of NK cells within solid tumors.

To address these formidable challenges, innovative strategies that enhance both the delivery and functional resilience of NK cells are imperative. Adipose‐derived mesenchymal stem cells (SCs) have attracted significant attention as a solution, due to their intrinsic tumor tropism.^[^ ^13^ ^]^ Driven by the CXCR4/SDF‐1 axis, ADSCs are naturally attracted to the hypoxic and inflamed niches characteristic of solid tumors.^[^ ^14^, ^15^ ^]^ This property makes them excellent candidates as cellular vehicles for the targeted delivery of NK cells. Furthermore, interleukin‐15 (IL‐15), a critical cytokine for NK cell survival and activation, has shown therapeutic potential but is hampered by systemic toxicity and short half‐life when administered conventionally.^[^ ^16^ ^]^ Localized, sustained IL‐15 delivery via engineered SCs could circumvent these limitations while synergizing with NK cell activity through paracrine signaling.

**Figure 1:**
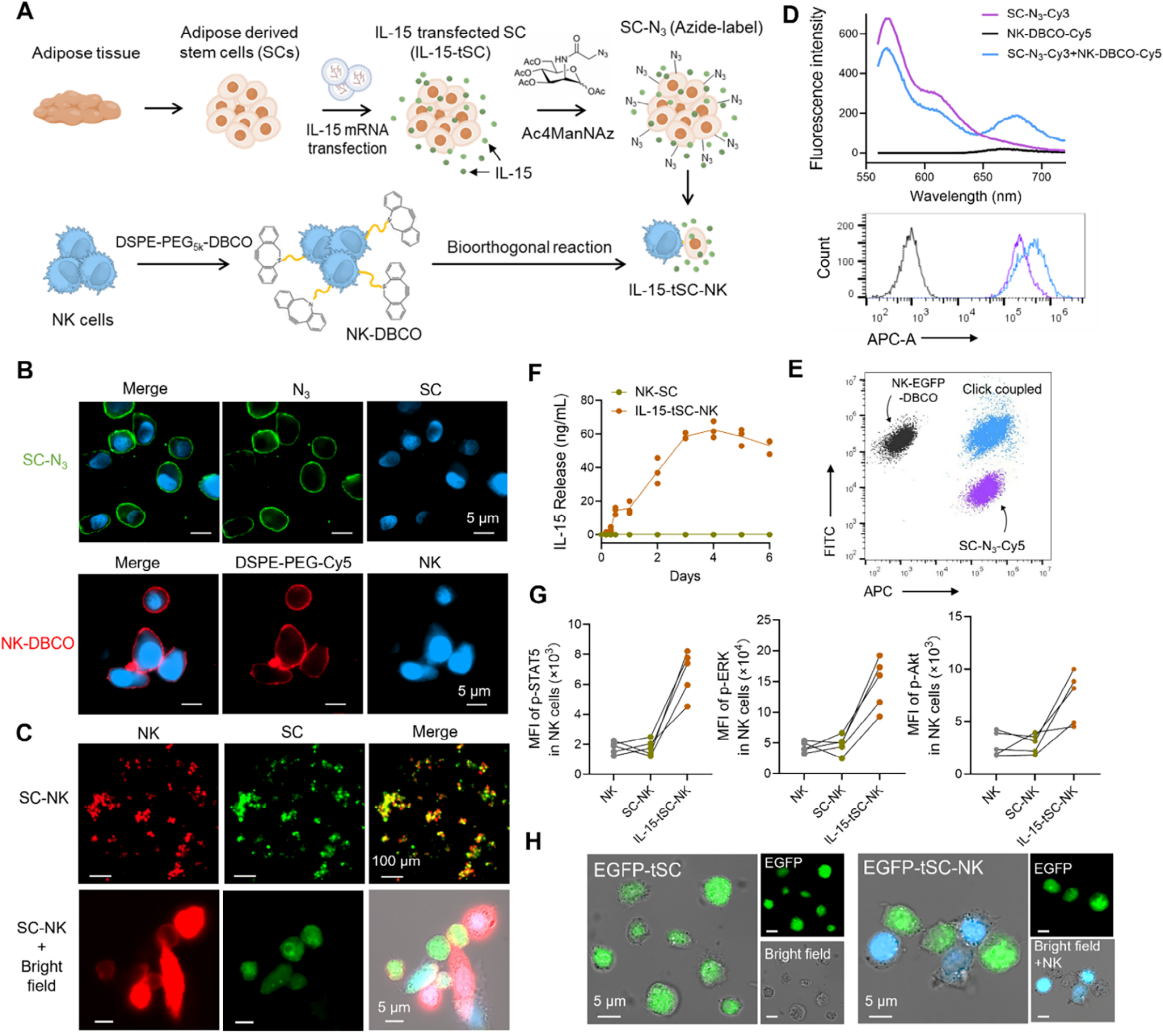
Characterization of engineered IL‐15‐expressing SC‐NK cell complexes via click chemistry. A) Schematic illustration of IL‐15‐transfected adipose‐derived mesenchymal stem cells (IL‐15‐tSC) via mRNA transfection, followed by azide‐functionalization using Ac_4_ManNAz metabolic labeling. NK cells modified with dibenzocyclooctyne (DBCO) conjugated phospholipid (DSPE‐PEG_5k_‐DBCO) were covalently linked to SC‐N_3_ cells through click reaction, generating IL‐15‐tSC‐NK complexes. B) Fluorescence microscopy confirming successful azide‐labeling of adipose‐derived mesenchymal stem cells and DBCO‐modification of NK cells. SCs were azide‐labeled and subsequently stained with DBCO‐Cy3 (green), while NK cells were modified with DSPE‐PEG‐Cy5 (red). Cell nuclei were stained with DAPI (blue). Scale bars, 5 µm. C) Representative fluorescence images demonstrating specific conjugation of NK cells to SC cells. Scale bars: 100 and 5 µm. D) (Top) Fluorescence emission spectra showing fluorescence resonance energy transfer (FRET) effect between Cy3‐labeled SC‐N_3_ and Cy5‐labeled NK‐DBCO upon conjugation. A spectral shift and intensity increase indicate energy transfer due to reduced intercellular distance. (Bottom) Flow cytometry analysis of the conjugates. The shift in Cy3 (SCs) signal toward NK cell population confirms membrane–membrane proximity and efficient SC–NK binding. E) Flow cytometry analysis showing click coupling between NK cells expressing EGFP and Cy5‐labeled SC. F) Release profiles of IL‐15 from IL‐15‐tSC‐NK complexes in 6 days. SC‐NK complex was presented as control. G) Flow cytometry quantification demonstrating the changes in phosphorylation of signaling proteins (p‐STAT5, p‐ERK, p‐Akt) in NK cells after interaction with IL‐15‐expressing SC cells. H) Fluorescence images showing the EGFP‐tSC‐NK complexes. Scale bars: 5 µm.

Herein, we designed a cell‐cell complex delivery system integrating click chemistry‐conjugated SC carriers with IL‐15 mRNA lipid nanoparticle (LNP) engineering to potentiate NK cells. By covalently linking NK cells to SCs via bioorthogonal click chemistry (DBCO‐azide), we aim to exploit SC‐mediated tumor homing for enhanced NK cell localization. Concurrently, transient IL‐15 expression by SCs through mRNA transfection is engineered to provide spatially controlled cytokine support, revitalizing NK cell effector functions within the TME. (**Figure** **1**A). To this end, we set out to test the following working hypothesis: spatially guided delivery of IL‐15–expressing stromal cell–NK cell complexes will i) improve intratumoral NK‐cell localization, ii) sustain NK‐cell cytotoxic function within an immunosuppressive TME, and iii) translate into superior antitumor efficacy that can be further potentiated by Galectin‐9 blockade.

## 2 Results

### 2.1 Construction of IL‐15‐tSC‐NK Complexes

First, IL‐15 mRNA LNP was efficiently transfected into SCs, followed by metabolic labeling using Ac_4_ManNAz, introducing azide functional groups onto the cell surface (SC‐N_3_). Concurrently, NK cells were functionalized with DBCO‐conjugated phospholipids (DSPE‐PEG‐DBCO). Subsequently, NK cells and SC‐N_3_ were successfully conjugated via click chemistry, generating stable IL‐15‐expressing SC‐NK complexes (IL‐15‐tSC‐NK, Figure 1A). Fluorescence microscopy confirmed the successful labeling of SCs with azide groups and NK cells with DBCO groups (Figure 1B). Merged fluorescence images clearly illustrated the successful attachment and colocalization of NK cells (red) with SCs (green), indicative of specific conjugation (Figure 1C). Further validation using fluorescence emission spectroscopy and flow cytometry demonstrated the dual fluorescence signals of the complex (Cy3 and Cy5), confirming the efficient formation of click chemistry‐mediated SC‐NK complexes (Figure 1D,E; Figure S1, Supporting Information). Functional assays revealed sustained and robust secretion of IL‐15 from the IL‐15‐tSC‐NK complexes over 6 days, compared to minimal IL‐15 release from non‐transfected control NK‐SC complexes (Figure 1F). Consistent with IL‐15 biological activity, NK cells conjugated with IL‐15‐expressing ADSCs exhibited significantly enhanced intracellular signaling, as indicated by increased phosphorylation levels of STAT5, ERK, and Akt, key molecules involved in NK cell proliferation, activation, and survival (Figure 1G).^[^ ^17^, ^18^ ^]^ Finally, microscopy imaging of EGFP‐expressing SCs showed stable complex formation with NK cells, reinforcing the feasibility and robustness of this conjugation strategy for further therapeutic applications (Figure 1H). Collectively, these findings confirm the construction of bioengineered IL‐15‐expressing SC‐NK platform with potential to enhance NK cell‐mediated antitumor immunity.

### 2.2 Enhanced Cytotoxicity and Immune Activation of IL‐15‐tSC‐NK Complexes

**Figure 2:**
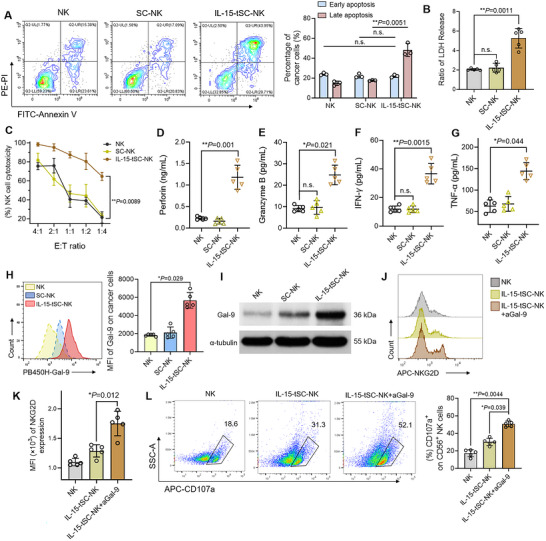
In vitro cytotoxicity and functional mechanism analyses of IL‐15‐tSC‐NK complexes. A) Flow cytometry analysis of tumor cell apoptosis after co‐culture with NK cells, SC‐NK, or IL‐15‐tSC‐NK complexes for 24 h. Apoptotic cells were defined as early apoptotic (Annexin V⁺/PI−) or late apoptotic (Annexin V⁺/PI⁺). B) Quantification of tumor cell lysis using lactate dehydrogenase (LDH) release assay (*n* = 5). C) Tumor cell viability under various effector‐to‐target (E:T) ratios assessed by CCK‐8 assay (*n* = 5). ELISA quantification of cytotoxic cytokines and effector molecules secreted by different NK formulations: perforin D), granzyme B E), IFN‐γ F), and TNF‐α (G) (*n* = 5). Flow cytometry H) and western blot I) analysis of galectin‐9 expression on tumor cells post‐treatment. Flow cytometry analysis of NKG2D J,K) and CD107a L) expression in NK, SC‐NK, and IL‐15‐tSC‐NK groups (*n* = 5). For the aGal‐9 group, anti‐Galectin‐9 antibody was administered 24 h after initial co‐culture with NK cells, and cells were harvested and analyzed after an additional 24‐h incubation (*n* = 5). Data are presented as means ± s.d. Statistical significance was assessed using one‐way ANOVA with multiple comparisons. *p < 0.05, **p < 0.01.

The in vitro cytotoxic effects and underlying mechanisms of IL‐15‐tSC‐NK were comprehensively evaluated. To ensure optimal functionality, IL‐15‐tSC‐NK complexes used in cytotoxicity assays were harvested and co‐cultured with tumor cells on day 2 post‐manufacturing, when IL‐15 secretion reached near‐peak levels (Figure 1F). This time point was selected based on time‐course analysis (Figure S1, Supporting Information), which showed that day 2 cells exhibited significantly higher cytotoxic activity than day 1 cells, and comparable potency to days 3 and 5 (Figure S2, Supporting Information). Flow cytometry analysis using Annexin V and propidium iodide (PI) staining demonstrated that cancer cells co‐cultured with IL‐15‐tSC‐NK exhibited significantly increased percentages of late apoptosis (Annexin V^+^ PI^+^) compared to cells treated with NK cells alone or SC‐NK complexes without IL‐15 modification (**Figure** **2**A). In line with these results, LDH release assays showed that IL‐15‐tSC‐NK complexes induced 2.6‐fold higher tumor cell cytotoxicity compared to NK cells (Figure 2B). To further quantify NK‐mediated cytotoxic capacity, killing efficiency was measured across various effector‐to‐target (E:T) cell ratios. At an E:T ratio of 1:1, IL‐15‐tSC‐NK complexes exhibited a 2.2‐fold increase in cytotoxicity compared to conventional NK cells and a 1.8‐fold increase compared to SC‐NK complexes (Figure 2C). Mechanistic investigations further revealed significant increases in the secretion of cytotoxic mediators, including perforin, granzyme B, IFN‐γ, and TNF‐α (Figure 2D–G) from IL‐15‐tSC‐NK complexes compared to NK or SC‐NK controls. The elevated cytokine profiles suggest markedly improved NK cell activation and effector function following conjugation with IL‐15‐expressing SCs. PD‐1 expression analysis showed comparable exhaustion profiles between IL‐15‐tSC‐NK and unmodified NK cells over time, indicating that the engineering approach does not significantly affect NK cell exhaustion (Figure S3, Supporting Information). Interestingly, co‐culture with IL‐15‐tSC‐NK complexes led to a pronounced upregulation of Gal‐9 expression on the surface of tumor cells (Figure 2H). This was further validated by western blot, which confirmed increased Gal‐9 protein levels in tumor cells following IL‐15‐tSC‐NK treatment (Figure 2I). This upregulation likely reflects a stress or immune escape response triggered by NK‐mediated cytotoxic pressure.^[^ ^19^, ^20^ ^]^ Since Gal‐9 has been implicated in the suppression of NK cell cytotoxicity via binding to receptors such as Tim‐3, its elevated expression may represent a potential resistance mechanism that limits prolonged NK activity.^[^ ^10^, ^21^ ^]^

To counteract this adaptive resistance mechanism, we assessed the impact of combining IL‐15‐tSC‐NK therapy with Gal‐9 blockade. Flow cytometric analysis demonstrated that co‐treatment with anti–Gal‐9 antibody significantly enhanced the expression of NKG2D, a key activating receptor on NK cells (Figure 2J,K), compared to IL‐15‐tSC‐NK treatment alone. Moreover, expression of the degranulation marker CD107a was further elevated under the combination treatment (Figure 2L), indicating enhanced NK cell degranulation and cytotoxic potential. These findings suggest that Gal‐9 blockade synergizes with IL‐15‐driven activation to further amplify NK cell effector functions and overcome tumor‐induced immunosuppression.

### 2.3 Synergistic Suppression of Orthotopic Lung Cancer by IL‐15‐tSC‐NK + Anti‐Gal‐9 Therapy

**Figure 3:**
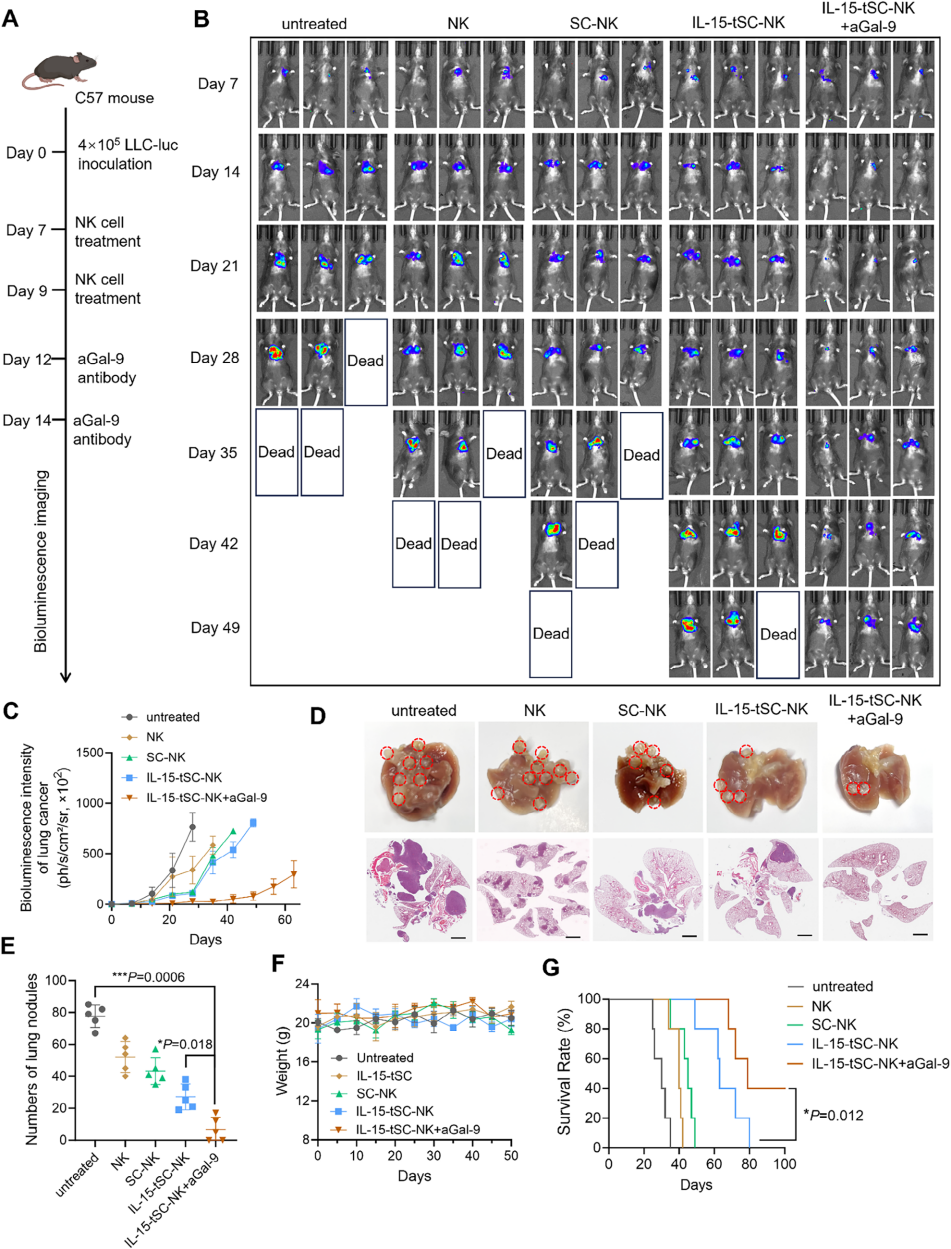
Therapeutic efficacy of IL‐15‐tSC‐NK combined with anti‐Galectin‐9 antibody (aGal‐9) in a mouse lung cancer model. A) Schedules for NK cell and aGal‐9 treatment regimen. C57BL/6 mice were inoculated intravenously with 4 × 10^5^ luciferase‐expressing Lewis lung carcinoma (LLC‐luc) cells on day 0. 1.2 × 10^6^ NK, SC‐NK, or IL‐15‐tSC‐NK were administered intravenously on days 7 and 9. The anti‐Gal‐9 antibody treatment was intraperitoneal injected on days 12 and 14. Tumor progression was monitored by bioluminescence imaging at indicated time points. B) Representative bioluminescence images illustrating lung cancer progression in a 49‐day period. C) Quantitative analysis of tumor growth shown by bioluminescence intensity. D) Representative images and histological analysis with H&E staining of lungs on day 20. Scale bars: 200 µm. E) Quantification of lung tumor nodules on day 25 (*n* = 6). F) Body weight measurements throughout the treatment (*n* = 6). G) Survival curves after tumor inoculation (*n* = 6). Data are presented as mean ± s.d. Statistical significance was calculated via one‐way ANOVA followed by Tukey's multiple comparison. *p < 0.05, ***p < 0.001.

To evaluate in vivo therapeutic efficacy of IL‐15‐tSC‐NK cells in combination with Gal‐9 blockade, we employed a murine model of lung cancer by intravenously injecting luciferase‐expressing Lewis lung carcinoma (LLC‐luc) cells into C57BL/6 mice. Treatment regimens included NK cells, SC‐NK cells, IL‐15‐tSC‐NK cells, and IL‐15‐tSC‐NK combined with aGal‐9 antibody, administered according to the schedule shown in **Figure** **3**A. Compared to NK cell treatment alone, SC‐NK cells exhibited moderately enhanced antitumor activity, as indicated by reduced tumor signals in bioluminescence imaging. This improvement may be attributed to SC‐mediated enhancement of NK cell tumor‐targeting ability, allowing for more efficient homing and interaction with tumor lesions. IL‐15‐tSC‐NK therapy further amplified tumor suppression, significantly inhibiting lung tumor progression compared to both NK and SC‐NK groups, highlighting the critical role of IL‐15 in potentiating NK cell functionality. Notably, the addition of aGal‐9 antibody synergistically enhanced the therapeutic outcome, resulting in a more pronounced and sustained reduction in tumor burden with the lowest tumor signals (Figure 3B,C; Figure S4, Supporting Information). Importantly, no significant changes in body weight were observed throughout the treatment period (Figure 3D), suggesting that the therapies were well tolerated and systemically safe. Consistent with the tumor inhibition findings, survival analysis (Figure 3E) demonstrated significantly prolonged survival in mice receiving the combination therapy compared to all other groups. Furthermore, histological analysis of lung sections (Figure 3F) and pulmonary tumor nodule count (Figure 3G) revealed that IL‐15‐tSC‐NK + aGal‐9 treatment markedly reduced tumor lesions, confirming its superior efficacy in reducing metastatic tumor burden. Collectively, these results demonstrate that IL‐15‐tSC‐NK, especially when combined with Gal‐9 blockade, elicit potent, sustained antitumor responses with minimal systemic toxicity, offering a promising therapeutic strategy against orthotopic lung cancer.

### 2.4 Therapeutic Efficacy in a Patient‐Derived Xenograft Ovarian Cancer Model

**Figure 4:**
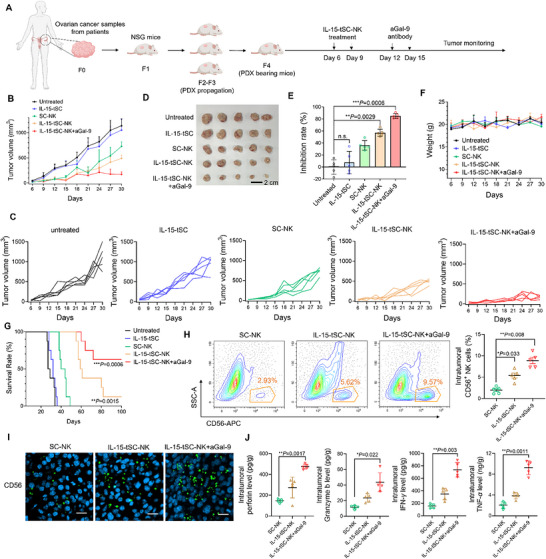
Therapeutic effects of IL‐15‐tSC‐NK combined with anti‐Galectin‐9 antibody in a patient‐derived xenograft (PDX) ovarian cancer model. A) Schematic illustration of the experimental workflow, including engraftment of human ovarian cancer tissue into NSG mice and administration of NK‐based therapies. Overall B) and individual C) tumor volume growth curves of NSG mice receiving various NK cell treatment and aGal‐9 antibody therapy. D) Representative images of excised PDX tumors from all groups on day 28. E) Quantitative analysis of tumor inhibition rate. F) Body weight monitoring throughout the experiment. G) Survival curves of mice after PDX cancer model establishment (*n* = 6). H) Flow cytometry analysis of human NK cell infiltration in tumor tissues from different treatment groups, quantified by the percentage of CD45⁺CD56⁺ cells (*n* = 5). I) Immunofluorescence staining of tumor sections for human CD56⁺ NK cell infiltration (red); nuclei counterstained with DAPI (blue). Scale bar: 50 µm. J) Intratumoral levels of perforin, granzyme B, IFN‐γ in mice treated with various NK‐based formulations (*n* = 5). Data are presented as means ± s.d. Statistical significance was assessed using one‐way ANOVA with multiple comparisons. *p < 0.05, **p < 0.01, ***p < 0.001.

Having demonstrated the antitumor potential of IL‐15‐tSC‐NK therapy in murine models, we next sought to validate the translational relevance of this strategy using a patient‐derived xenograft (PDX) model of ovarian cancer in immunodeficient NSG mice. Human ovarian tumor tissues were engrafted and propagated through serial passaging, and treatment was initiated following the tumor establishment. Mice were randomized into five treatment groups: untreated, IL‐15‐tSC, SC‐NK, IL‐15‐tSC‐NK, and IL‐15‐tSC‐NK combined with anti‐Gal‐9 antibody (**Figure** **4**A). We did not include the NK‐alone group in this study, as our previous lung cancer model showed that NK cell monotherapy, due to insufficient tumor infiltration and functional impairment, failed to achieve satisfactory therapeutic efficacy compared to the SC‐NK group. Moreover, preclinical reports have demonstrated that adoptively transferred NK cells without additional modifications display minimal therapeutic activity against solid tumors.^[^ ^22^, ^23^ ^]^ Tumor volume measurements revealed that IL‐15‐tSC treatment alone had no discernible therapeutic impact, with tumor progression comparable to the untreated group (Figure 4B,C). SC‐NK treatment moderately delayed tumor growth. In contrast, IL‐15‐tSC‐NK therapy resulted in a marked and consistent reduction in tumor burden, underscoring the importance of IL‐15 in sustaining NK cell functionality within the tumor microenvironment. Notably, the combination of IL‐15‐tSC‐NK and aGal‐9 achieved the most pronounced tumor suppression across all groups, maintaining minimal tumor volumes throughout the 28‐day observation period. At day 28, excised tumors clearly reflected treatment efficacy, with the smallest tumors observed in the IL‐15‐tSC‐NK+aGal‐9 group (Figure 4D). Quantitative analysis confirmed a significantly elevated tumor inhibition rate in this group (≈85.2%), which was statistically superior to both SC‐NK and IL‐15‐tSC‐NK monotherapies (Figure 4E). Throughout the treatment period, body weight remained stable in all mice (Figure 4F). Survival analysis further supported the therapeutic benefit of the dual treatment: mice receiving IL‐15‐tSC‐NK+aGal‐9 experienced the longest survival, with a survival period exceeding 100 days (Figure 4G). To investigate the underlying mechanisms contributing to the superior antitumor efficacy, we analyzed NK cell infiltration within tumor tissues. Flow cytometry analysis showed a significant increase in CD45⁺CD56⁺ NK cells in the IL‐15‐tSC‐NK+aGal‐9 group compared to SC‐NK complexes and IL‐15‐tSC‐NK monotherapy. Specifically, IL‐15‐tSC‐NK treatment resulted in a 3.2‐fold increase in CD45⁺CD56⁺ NK cell infiltration relative to the NK group, and a 1.7‐fold increase compared to the SC‐NK group (Figure 4H). These findings were corroborated by immunofluorescence staining, which revealed dense intratumoral CD56⁺ NK cell presence in the combination therapy group (Figure 4I), suggesting that both improved homing and enhanced persistence contributed to the therapeutic outcome. Intratumoral cytokine analysis revealed that IL‐15‐tSC‐NK treatment significantly elevated the secretion of perforin, granzyme B, and IFN‐γ compared to SC‐NK complex, indicating enhanced NK cell activation within the tumor microenvironment (Figure 4J).

Collectively, these results demonstrate that IL‐15‐tSC‐NK therapy, particularly when combined with Galectin‐9 blockade, elicits potent antitumor immunity in a clinically relevant ovarian cancer PDX model. Through enhancing NK cell infiltration, sustaining effector function, and promoting local immune activation, this dual approach offers a promising and well‐tolerated strategy to overcome immune resistance in solid tumors.

### 2.5 IL‐15‐tSC‐NK Facilitates Targeted Tumor Infiltration and Potentiates Intratumoral Immune Activation

**Figure 5:**
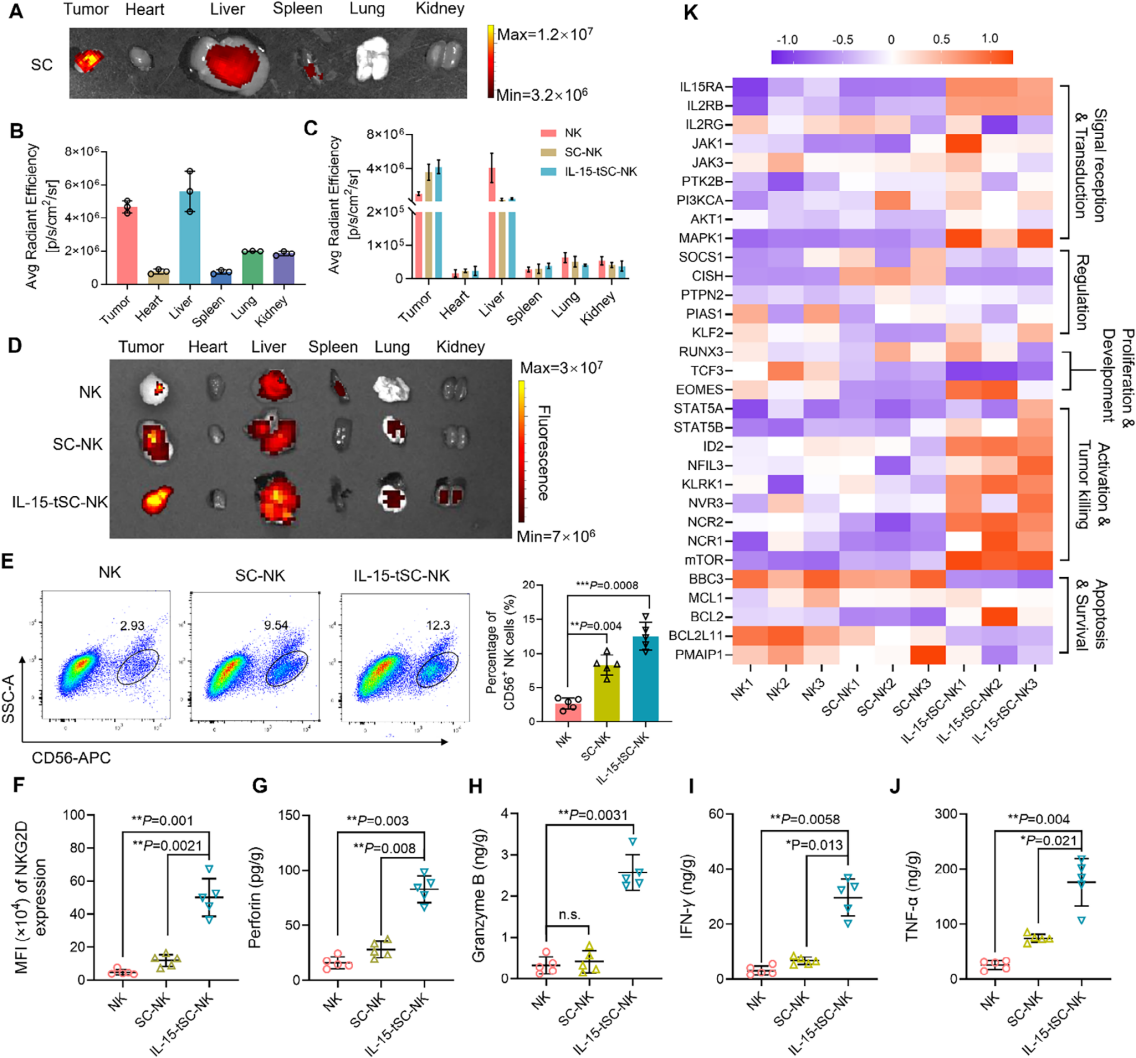
Mechanistic investigation of IL‐15‐tSC‐NK mediated antitumor responses and immunomodulation. A) In vivo biodistribution of DiR‐labeled SCs in tumor‐bearing mice, imaged at 24 h post‐injection. B) Quantification of SC fluorescence intensity in major organs (*n* = 3). C,D) Quantitative analysis and biodistribution of DiR‐labeled NK cells in tumor‐bearing mice following systemic administration of NK, SC‐NK, or IL‐15‐tSC‐NK. E) Flow cytometry plots showing the frequency of intratumoral (CD3−CD56⁺) NK cells (*n* = 5). F) Quantification of NKG2D expression in NK cells based on flow cytometry analysis (*n* = 5). Intratumoral levels of perforin (G), granzyme B (H), IFN‐γ (I), and TNF‐α (J) in tumors treated with various NK‐based formulations (*n* = 5). K) Heatmap showing the relative expression of intracellular pathway signaling in NK cells (*n* = 3). Data are presented as means ± s.d. Statistical significance was assessed using one‐way ANOVA with multiple comparisons. *p < 0.05, **p < 0.01, ***p < 0.001.

To elucidate the mechanisms underlying the potent antitumor efficacy of IL‐15‐tSC‐NK, we first assessed the in vivo biodistribution of stem cells (SCs), which serve as carriers in this delivery platform. In tumor‐bearing mice, fluorescence imaging revealed that systemically administered SCs exhibited preferential accumulation within tumor tissues, highlighting their intrinsic tumor‐homing capacity (**Figure** **5**A). Similarly, DiR‐labeled NK cells delivered via IL‐15‐tSC‐NK complexes showed markedly enhanced localization to tumor sites compared to free NK cells, suggesting that SC‐mediated delivery synergized with IL‐15 signaling to improve NK cell retention within the tumor microenvironment (Figure 5B). Flow cytometric analysis of resected tumor tissues revealed significantly increased infiltration of CD3^‐^CD56⁺ NK cells in mice treated with SC‐NK and IL‐15‐tSC‐NK complex compared to NK monotherapy (Figure 5C,D). Notably, IL‐15‐tSC‐NK treatment yielded the highest NK cell frequency, indicating that stem cell conjugation, combined with paracrine IL‐15 signaling, markedly enhances NK cell trafficking and retention within the tumor microenvironment (Figure 5E). This improved accumulation likely reflects the synergistic contributions of SC‐mediated tumor homing and IL‐15‐driven local survival and expansion of NK cells.

Next, we examined whether the infiltrating NK cells exhibited functional activation within the tumor microenvironment. Flow cytometry revealed that IL‐15‐tSC‐NK complex exhibited significantly elevated surface expression of the activating receptor NKG2D compared to both NK and SC‐NK groups (Figure 5F). Measurement of key effector molecules revealed that tumors from SC‐NK–treated mice showed elevated levels of perforin, granzyme B, IFN‐γ, and TNF‐α, suggesting that co‐localization with SCs partially restored NK cytotoxic functionality (Figure 5G–J). Remarkably, IL‐15‐tSC‐NK treatment further amplified this response, resulting in the highest expression levels of all four mediators. This indicates that localized, cell‐bound IL‐15 delivery not only promotes NK cell retention but also enhances their cytotoxic potency within the immunosuppressive tumor milieu.

To gain mechanistic insights into intracellular signaling events responsible for these functional gains, we performed proteomic profiling of tumor‐infiltrating NK cells. Heatmap analysis of differentially expressed immune‐related proteins revealed that IL‐15‐tSC‐NK treatment induced upregulation of key signaling nodes associated with immune activation, including STAT5, PI3K‐Akt, and MAPK pathways, as well as increased expression of proteins involved in signal reception/transduction, regulation, proliferation/development, apoptosis/survival, and tumor killing (Figure 5K). Notably, proteins associated with NK cell exhaustion or inhibitory signaling remained unchanged or were downregulated, suggesting a favorable shift in immune signaling balance. These protein‐level changes reflect a comprehensive rewiring of NK intracellular pathways toward enhanced cytotoxicity, survival, and resistance to immunosuppressive cues within the tumor microenvironment.^[^ ^24^ ^]^ These findings demonstrate that IL‐15‐tSC‐NK therapy promotes not only increased NK cell infiltration and cytokine production but also profound proteomic reprogramming that reinforces their antitumor function and persistence within solid tumors.

### 2.6 Transcriptomic Profiling Reveals Immune Activation Signatures Within IL‐15‐tSC‐NK Cells

**Figure 6:**
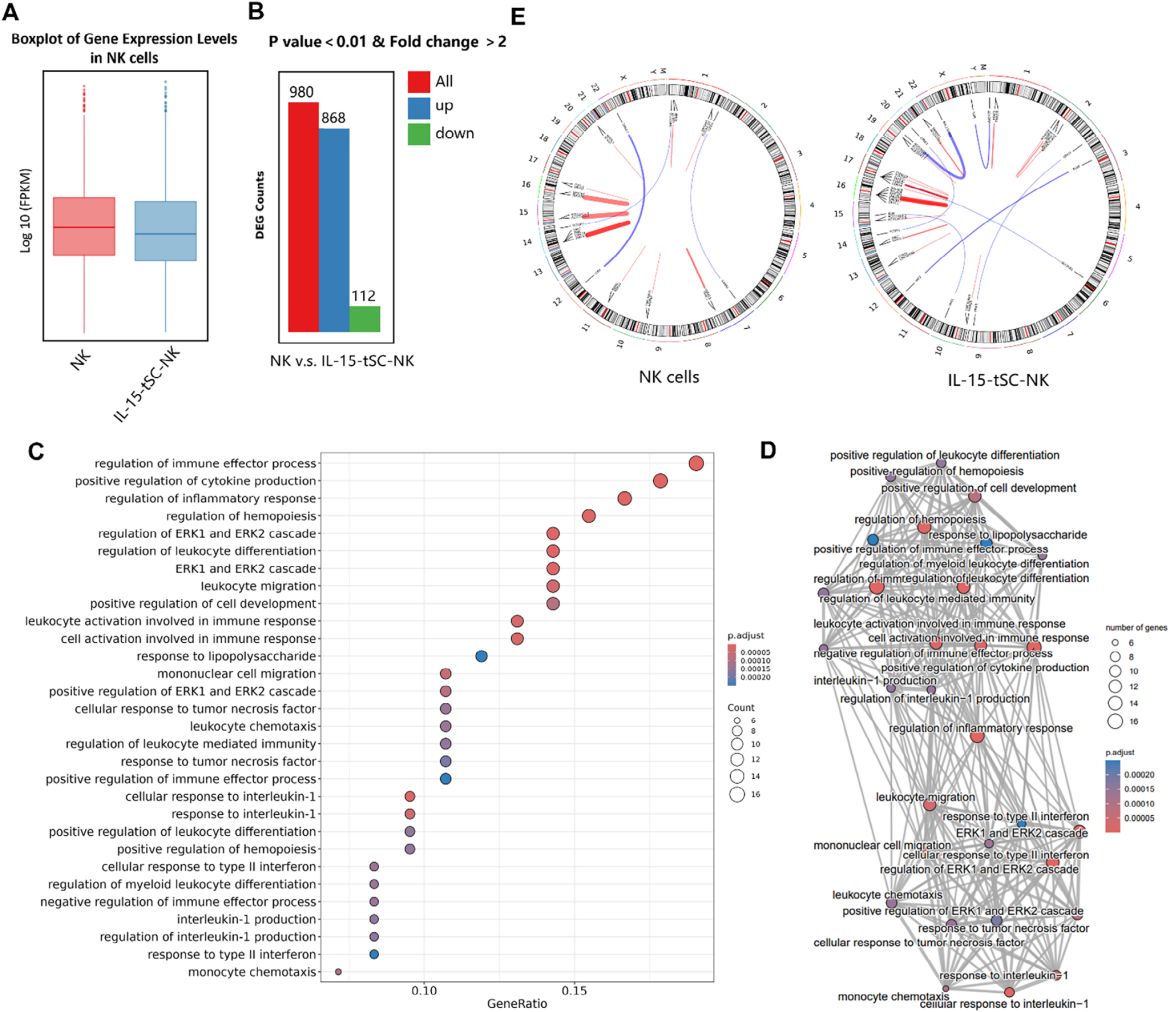
Transcriptomic analysis of NK and IL‐15‐tSC‐NK. A) Box plot showing normalized gene expression levels (log_10_ FPKM). B) Bar chart displaying the number of differentially expressed genes identified in each comparison group, including upregulated (blue), downregulated (green), and all significant (red) genes. C) Gene Ontology (GO) enrichment analysis of differentially expressed genes (DEGs) from NK cells isolated from tumor tissues following treatment. GeneRatio indicates the proportion of genes enriched in each GO term. RNA‐seq data were obtained from magnetically isolated NK cells from tumor tissues to ensure the cell‐type specificity of the transcriptomic profiling. D) GO network showing the relationship and clustering among enriched immune regulatory pathways. E) Circos plots showing chromosomal locations and expression changes of representative DEGs between NK and IL‐15‐tSC‐NK groups. Red and blue lines represent upregulated and downregulated genes, respectively.

To investigate the transcriptional consequences of IL‐15‐tSC‐NK, we performed RNA sequencing on tumor‐infiltrating NK cells. Global gene expression profiles, measured by log_10_‐transformed FPKM values, showed no significant difference in distribution between IL‐15‐tSC‐NK and NK cells, indicating that the engineering strategy did not broadly alter the transcriptomic landscape (**Figure** **6**A). Instead, functional enhancements observed upon treatment are likely driven by specific changes in immune‐regulatory gene expression.

Differential expression analysis identified 980 significantly altered genes between the two groups (*p* < 0.01, fold change > 2), including 868 upregulated and 112 downregulated transcripts in IL‐15‐tSC‐NK cells (Figure 6B). Gene Ontology (GO) enrichment analysis of these DEGs indicated significant enrichment of immune‐related pathways, such as regulation of immune effector processes, cytokine production, and ERK1/2 signaling cascades (Figure 6C). These enrichment results suggest transcriptional trends consistent with enhanced immune activity. GO network visualization further revealed dense clustering among the enriched terms, indicating coordinated upregulation of immune activation programs (Figure 6D). To further characterize the scope of transcriptomic alterations, we generated circos plots mapping the chromosomal distribution of representative DEGs. As shown in Figure 6E, engineered IL‐15‐tSC‐NK exhibited widespread gene expression changes across multiple genomic loci, reflecting the global impact of in vivo tumor exposure and treatment modulation on their transcriptional state. In addition, network plot illustrating significantly enriched KEGG pathways among DEGs in IL‐15‐tSC‐NK compared to NK cells. Engineered cell complexes showed marked enrichment of multiple immune‐related pathways, including JAK‐STAT signaling, cytotoxicity, and chemokine signaling, highlighting their potential role in enhancing NK cell function (Figure S5, Supporting Information).

Together, these transcriptomic results demonstrate that while global transcriptional activity remains stable, IL‐15‐tSC‐NK treatment enhanced the expression of immune effector genes and signaling pathways, supporting its role in reprogramming NK cells for improved antitumor function.

### 2.7 In Vivo Biosafety Evaluation of IL‐15‐tSC‐NK + aGal‐9 Therapy

**Figure 7:**
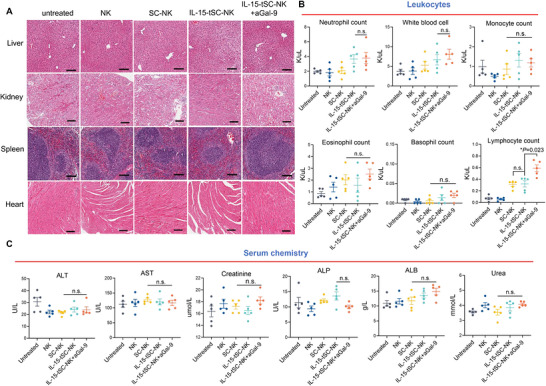
Biosafety evaluation of IL‐15‐tSC‐NK combined with anti‐Galectin‐9 antibody in vivo. A) Representative hematoxylin and eosin H&E staining images of major organs (liver, kidney, spleen, heart) collected from mice 28 days after treatment with NK, SC‐NK, IL‐15‐tSC‐NK, or IL‐15‐tSC‐NK + aGal‐9. Scale bars: 200 µm. B) Peripheral leukocyte counts including total white blood cells, neutrophils, monocytes, eosinophils, basophils, and lymphocytes were analyzed by automated hematology analyzer. C) Serum biochemical parameters including ALT, AST, creatinine, ALP, ALB, and urea were measured to assess hepatic and renal function across treatment groups. Data are presented as mean ± SD. Statistical significance was analyzed using one‐way ANOVA analysis. *p < 0.05.

To assess the systemic safety of IL‐15‐tSC‐NK therapy and its combination with aGal‐9 antibody, histopathological and biochemical analyses were performed in tumor‐free mice following treatment. H&E staining of major organs, including liver, kidney, spleen, and heart, collected on day 28 post‐treatment, revealed no signs of tissue damage or inflammatory infiltration in any of the treatment groups (**Figure** **7**A). The histological morphology remained intact and comparable to untreated controls, indicating no observable organ toxicity associated with NK, SC‐NK, IL‐15‐tSC‐NK, or IL‐15‐tSC‐NK+aGal‐9 administration. Further evaluation of peripheral blood leukocyte composition showed that most immune cell subsets, including total white blood cells, neutrophils, monocytes, eosinophils, and basophils, were unaffected by treatment (Figure 7B). Notably, a mild but statistically significant increase in lymphocyte counts was observed in the IL‐15‐tSC‐NK+aGal‐9 group compared to the untreated group, suggesting possible immune activation without systemic inflammation. IL‐15‐tSC‐NK+aGal‐9 treatments had no significant effect on mean platelet volume or platelet count in mice (Figure S6, Supporting Information). In addition, serum chemistry analysis was conducted to evaluate hepatic and renal function.^[^ ^25^ ^]^ No significant changes were detected in levels of alanine aminotransferase (ALT), aspartate aminotransferase (AST), creatinine, alkaline phosphatase (ALP), albumin (ALB), or urea across all groups (Figure 7C). These results demonstrated that IL‐15‐tSC‐NK complex, alone or in combination with Gal‐9 blockade, does not induce overt toxicity in major organs and maintains normal hematological and biochemical parameters, supporting its biosafety for in vivo applications

## 3 Discussion and Conclusion

Overcoming the spatial and functional limitations of NK cell therapies in solid tumors remains a critical hurdle in the field of cancer immunotherapy. In this study, we developed a dual‐engineered cell–cell delivery platform, IL‐15‐tSC‐NK, which integrates mesenchymal stem cell‐guided NK delivery with transient IL‐15 support to enhance intratumoral accumulation and effector function of NK cells. Furthermore, we demonstrated that the combination of this platform with Galectin‐9 blockade—an immunosuppressive checkpoint highly enriched in the tumor microenvironment—achieves potent, durable antitumor efficacy in both murine and patient‐derived xenograft models of solid tumors. From a translational perspective, the clinical feasibility of this strategy could be further supported by the flexibility in sourcing SCs. In principle, SCs may be derived from autologous adipose tissue, providing a personalized and immunologically compatible vehicle for NK cell delivery. Alternatively, allogeneic SCs from established cell banks could offer a more standardized and scalable solution for broader clinical deployment.^[^ ^26^, ^27^ ^]^ Given the low immunogenicity and well‐documented safety profile of SCs, as well as the increasing availability of GMP‐compliant expansion protocols, both sourcing strategies may be compatible with clinical manufacturing pipelines.^[^ ^28^, ^29^ ^]^

Mechanistically, SCs served not only as tumor‐homing carriers that significantly improved NK cell trafficking, but also as local “cytokine depots” to provide spatially restricted IL‐15 signals. This local IL‐15 expression proved critical for maintaining NK cell cytotoxicity, proliferation, and persistence within the suppressive TME.^[^ ^30^, ^31^ ^]^ Compared to SC‐NK or IL‐15‐tSC monotherapies, the IL‐15‐tSC‐NK complex markedly enhanced intratumoral NK cell infiltration, increased secretion of cytotoxic effectors including perforin, granzyme B, and IFN‐γ, and exhibited robust tumor control in vivo. Interestingly, we observed that tumor cells upregulated surface Gal‐9 expression following NK cell engagement, likely as an adaptive immune escape mechanism in response to cytotoxic stress. Given the known role of Gal‐9 in inducing NK cell dysfunction through TIM‐3 ligation, we hypothesized that its blockade could further potentiate NK cell activity.^[^ ^32^ ^]^ Indeed, co‐treatment with an anti‐Gal‐9 antibody synergistically enhanced IL‐15‐tSC‐NK‐mediated antitumor effects, leading to nearly complete tumor regression in the PDX model, significantly prolonged survival, and superior immune activation. This combinatorial approach effectively circumvented both physical delivery barriers and molecular immunosuppressive signals, achieving a level of efficacy not attainable with either strategy alone.

The NSG‐based PDX model employed in this study was selected for its ability to support the stable engraftment of human ovarian tumors and to permit functional evaluation of adoptively transferred human NK cells without interference from endogenous murine lymphocytes. The lack of functional T cells, B cells, and NK cells in NSG mice ensures that the antitumor effects observed can be specifically attributed to the administered IL‐15‐tSC‐NK complexes. However, this model cannot capture immune cell interactions such as NK–T cell cross‐talk, antigen presentation, or regulatory feedback mechanisms that are present in patients. This difference is a recognized limitation of NSG‐based xenograft models and should be considered when interpreting NK cell–mediated effects in vivo.

It is worth noting that an unmodified NK cell group was not included in the PDX study, as numerous prior reports and our own preliminary data have demonstrated minimal efficacy of NK monotherapy in solid tumors due to poor tumor‐homing and rapid functional exhaustion. Our design intentionally focused on testing strategies that directly address these limitations through spatial targeting and immunomodulation. Collectively, this study establishes a modular and versatile platform for engineering functionally enhanced NK cells using stem cell carriers and cytokine programming. By simultaneously addressing NK cell delivery and resilience within the TME, the IL‐15‐tSC‐NK system—particularly when combined with Gal‐9 checkpoint blockade—offers a promising blueprint for next‐generation NK cell immunotherapy in solid tumors. Moreover, combining this approach with immune checkpoint inhibitors, chemotherapeutics, or novel metabolic modulators may offer synergistic benefits by addressing complementary mechanisms of tumor immune evasion.

While our findings demonstrate the therapeutic promise of IL‐15–expressing SC–NK complexes, several translational challenges remain. First, large‐scale manufacture of dual‐engineered cells will require closed, GMP‐compliant workflows for: i) rapid enrichment and expansion of donor‐ or patient‐derived MSCs and ii) reproducible mRNA–LNP transfection. Automated bioreactor culture systems and in‐line sterility testing—already used for CAR T manufacturing—should facilitate this scale‐up, but process optimization and cost‐of‐goods analyses will be needed before clinical deployment. Second, we note that while MSCs offer a clinically relevant and scalable source for engineering, variations in cell activity and transfection efficiency may result in inconsistent IL‐15 expression. Therefore, precise monitoring and control of IL‐15 levels will be critical for ensuring safety and efficacy in future clinical applications. Addressing these questions will be essential for translating the current proof‐of‐concept into a viable, off‐the‐shelf NK‐cell immunotherapy.

## 4 Experimental Section

### Preparation of IL‐15‐tSC‐NK Complexes

Adipose‐derived mesenchymal stem cells were transfected with IL‐15 mRNA (1 µg per 10^6^ cells) through lipid nanoparticle delivery to obtain IL‐15‐expressing mesenchymal stem cells (IL‐15‐tSCs). To introduce azide groups, 1 × 10^6^ IL‐15‐tSCs were metabolically labeled with 50 µm Ac_4_ManNAz (Sigma–Aldrich) for 48 h to generate azide‐functionalized cells (SC‐N_3_). NK cells were incubated with 10 µm dibenzocyclooctyne (DBCO, MCE)‐conjugated phospholipid (DSPE‐PEG_5000_‐DBCO) for 1 h at 37 °C to obtain DBCO‐modified NK cells (NK‐DBCO). Subsequently, click chemistry was performed by co‐incubating SC‐N_3_ and NK‐DBCO at a 1:1 ratio (5 × 10^5^ cells each) in serum‐free medium for 1 h at 37 °C to generate IL‐15‐tSC‐NK complexes. To verify azide modification and click‐mediated coupling, SC‐N_3_ and NK‐DBCO were labeled with fluorescent probes (e.g., DSPE‐PEG_5000_‐Cy5). Cells were fixed with 4% paraformaldehyde and stained with DAPI (10 µg mL^−1^, Beyotime) for nuclear visualization. Samples were imaged using confocal microscopy. Co‐localization of SC and NK cells in merged fluorescence channels confirmed successful complex formation. The fluorescence spectra of SC‐N_3_‐Cy3, NK‐DBCO‐Cy5, and SC‐N_3_‐Cy3‐NK‐DBCO‐Cy5 were measured using a flow cytometer. Click‐conjugated SC‐NK complexes were further analyzed by flow cytometry. NK cells expressing EGFP and SCs labeled with Cy5 were co‐incubated and analyzed on an APC and FITC channel to confirm dual labeling. To assess cytokine release, IL‐15‐tSC‐NK and NK‐SC complexes were cultured in six‐well plates for up to 6 days (1 × 10^6^ cells per well). At indicated time points (0–6 days), culture supernatants were collected and IL‐15 concentration was measured using a mouse IL‐15 ELISA kit (Invitrogen). To evaluate downstream signaling, NK cells were co‐cultured with SC or IL‐15‐tSC for 24 h, harvested, and fixed/permeabilized using a commercial intracellular staining kit. Cells were stained with antibodies against p‐STAT5 (Abcam, ab278764), p‐ERK (Abcam, ab278538), and p‐Akt (Abcam, ab278565). Mean fluorescence intensity was quantified by flow cytometry (BD LSRFortessa). To visualize NK‐SC complex formation, Hochest 33342‐labeled NK cells were co‐cultured with EGFP‐tSCs, fixed with 4% paraformaldehyde, and mounted on slides. These cell complexes were imaged by fluorescence microscopy. Merged images indicated the localization of NK and SC components.

### In Vitro Cytotoxicity and Functional Analysis of IL‐15‐tSC‐NK Complexes

LLC tumor cells were seeded in 24‐well plates (1 × 10^5^ cells per well) and allowed to adhere overnight. The following day, NK cells, SC‐NK, or IL‐15‐tSC‐NK complexes were added at a 2:1 effector‐to‐target (E:T) ratio and co‐cultured for 24 h. Notably, the SC‐NK and IL‐15‐tSC‐NK complexes were used for co‐culture on day 2 post‐manufacturing, based on IL‐15 secretion kinetics (Figure 1F). At this time point, IL‐15‐tSC‐NK cells exhibited near‐peak IL‐15 release, which was found to be optimal for enhancing NK cell activation and antitumor functionality. This ensured that NK cells were in a maximally functional state during the 24–48 h co‐culture period with tumor cells. Cells were harvested and stained with Annexin V‐FITC and propidium iodide (PI) following the manufacturer's instructions. Flow cytometry was performed using a BD LSRFortessa, and early (Annexin V⁺/PI−) and late (Annexin V⁺/PI⁺) apoptotic cells were quantified. To evaluate tumor cell lysis, the LDH release assay was performed using a commercial LDH Cytotoxicity Assay Kit (Beyotime). Briefly, LLC cells were co‐incubated with NK, SC‐NK, or IL‐15‐tSC‐NK at an E:T ratio of 2:1 for 24 h in 96‐well plates. Supernatants were collected, and LDH activity was measured by absorbance at 490 nm. Cytotoxicity under different E:T ratios (4:1, 2:1, 1:1, 1:2, and 1:4) was assessed with LLC‐Luc cells and quantified by bioluminescence intensity using a microplate reader after incubation with D‐luciferin substrate, reflecting the viability of remaining cells. Supernatants from co‐cultures were collected after 24 h and analyzed using ELISA kits to quantify secreted levels of perforin (liankebio), granzyme B (liankebio), IFN‐*γ* (Invitrogen), and TNF‐*α* (Invitrogen). Assays were performed according to the manufacturers’ protocols, and absorbance was read at 450 nm.

To investigate the Galectin‐9 Expression in tumor cells LLC cells were treated with NK, SC‐NK, or IL‐15‐tSC‐NK cells for 24 h. For flow cytometry, cells were harvested, fixed, and stained with BV421‐conjugated anti‐galectin‐9 antibody (BioLegend 136119). Data were acquired on a flow cytometer. For western blotting, total protein was extracted using RIPA lysis buffer (Sigma–Aldrich), separated by SDS‐PAGE, and transferred to PVDF membranes. Membranes were incubated with anti‐galectin‐9 (Abcam, ab306119) and anti‐*β*‐actin (Abcam, ab8226) antibodies, followed by HRP‐conjugated secondary antibodies (Invitrogen) and detection using ECL substrate. For NKG2D and CD107a detection, tumor cells were co‐cultured with NK or IL‐15‐tSC‐NK for 24 h. To evaluate the effect of Galectin‐9 blockade, anti‐Gal‐9 antibody (10 µg mL^−1^, R&D Systems) was added to the culture medium 24 h after the initial NK cell treatment, and the cells were incubated for an additional 24 h. Following incubation, cells were stained with APC‐conjugated anti‐NKG2D (BioLegend, 130212) and APC‐conjugated anti‐CD107a (BioLegend, 121614) antibodies in staining buffer on ice for 30 min. Samples were then washed and analyzed by flow cytometry. For CD107a analysis, GolgiStop (BD Biosciences) was added during the final 4 h of the co‐culture period.

### In Vivo Antitumor Efficacy in an Orthotopic Lung Cancer Model

All animal experiments were approved by the Ethics Review Board of the First Affiliated Hospital of Anhui Medical University (Approval No. PJ‐2025‐03‐73) and conducted in accordance with institutional guidelines for the care and use of laboratory animals. C57BL/6 mice (6–8 weeks old, female) were intravenously inoculated via tail vein with 4 × 10⁵ luciferase‐expressing Lewis lung carcinoma cells (LLC‐Luc) suspended in 100 µL PBS on day 0 to establish an orthotopic lung cancer model. On days 7 and 9 post‐tumor inoculation, mice received intravenous injection of 2 × 10⁶ NK cells, SC‐NK, or IL‐15‐tSC‐NK complexes (in 100 µL PBS per mouse). For Galectin‐9 blockade, mice in the combination treatment group were intraperitoneally injected with anti‐Galectin‐9 antibody (aGal‐9, 200 µg per mouse, BioXcell) on days 12 and 14. Control mice received PBS injections. To monitor tumor progression, mice were injected intraperitoneally with D‐luciferin (150 mg kg^−1^, Yeasen) and anesthetized with isoflurane. Whole‐body bioluminescence signals were captured using an IVIS Spectrum imaging system (PerkinElmer) at days 7, 14, 21, 28, 35, 42, and 49. Luminescence intensity was quantified using Living Image software and expressed as total photon flux (photons/sec). Mice were observed daily for survival and weighed every 2–3 days throughout the study period. Endpoints were defined by >20% body weight loss or significant signs of distress.

On day 20, mice were euthanized, and lungs were harvested, photographed, and fixed in 4% paraformaldehyde. Visible surface tumor nodules were counted. Lung tissues were embedded in paraffin, sectioned, and stained with hematoxylin and eosin (H&E) for histological evaluation. Images were acquired with a brightfield microscope (Olympus), and tumor burden was qualitatively assessed.

### Establishment of Ovarian Cancer PDX Model

Fresh human ovarian tumor tissues were obtained with informed consent and cut into ≈30 mm^3^ fragments under sterile conditions. Female NSG mice (6–8 weeks old) were anesthetized and subcutaneously implanted with tumor tissue fragments into the right flank. Tumor engraftment was monitored every 3 days using caliper measurements (volume = 0.5 × length × width^2^), and mice were randomized into groups (*n* = 5) once tumors reached ≈50–100 mm^3^. Mice received intravenous injections of 2 × 10^6^ NK cells, SC‐NK, or IL‐15‐tSC‐NK complexes on days 6 and 9 post‐randomization. For the combination therapy group, mice were additionally treated with intraperitoneal injections of anti‐Galectin‐9 antibody (aGal‐9, 200 µg per mouse; BioXcell) on days 12 and 15. Control mice received equivalent volumes of PBS. Body weights were recorded every 3 days. Mice were monitored daily, and survival curves were plotted using the Kaplan–Meier method. Mice were euthanized upon reaching ethical endpoints (tumor volume >1200 mm^3^ or significant distress). Then tumors were excised, minced, and enzymatically digested into single‐cell suspensions using collagenase IV and DNase I. Cells were stained with APC‐conjugated anti‐human CD56 (BioLegend 981204) and FITC‐conjugated anti‐human CD45 antibodies (BioLegend 368508) for 30 min on ice, followed by washing and analysis using a flow cytometer. Tumor tissues were embedded in OCT compound and snap‐frozen in liquid nitrogen. Cryosections (10 µm) were prepared using a cryostat and mounted on glass slides. Sections were air‐dried, fixed with 4% paraformaldehyde for 10 min at room temperature, and washed with PBS. After blocking with 5% BSA in PBS for 1 h, sections were incubated overnight at 4 °C with anti‐human CD56 primary antibody (1:100, Abcam ab313779), followed by Alexa Fluor 594‐conjugated secondary antibody (1:500, Invitrogen) for 1 h at room temperature. Nuclei were counterstained with DAPI (1 µg mL^−1^), and images were captured using a confocal laser scanning microscope. Scale bar: 50 µm.

### Biodistribution and Mechanistic Evaluation of IL‐15‐tSC‐NK Treatment

To evaluate biodistribution, SCs were labeled with the near‐infrared dye DiR (Thermo Fisher) following the manufacturer's protocol. Labeled SCs were injected intravenously (1 × 10^6^ cells in 100 µL PBS) into tumor‐bearing mice. After 24 h, mice were sacrificed and major organs (tumor, heart, liver, spleen, lung, kidney) were harvested for ex vivo imaging using an IVIS Spectrum system (PerkinElmer). Fluorescence signals were quantified using Living Image software, and average radiant efficiency was calculated. For NK‐based therapies, NK, SC‐NK, or IL‐15‐tSC‐NK cells (2 × 10^6^ cells) were labeled with DiR and injected intravenously into mice. At 24 h post‐injection, organs were collected and imaged ex vivo using the same protocol. Fluorescence intensities were compared to assess tumor homing capacity.

### Tumor‐Infiltration Analysis

Tumor tissues were harvested 24 h after treatment, mechanically dissociated, and enzymatically digested with collagenase IV and DNase I to generate single‐cell suspensions. Cells were stained with fluorophore‐conjugated antibodies against human CD56 (APC, Abcam ab28335) and human CD3 (FITC, Abcam ab34275) for 30 min on ice. After washing, cells were analyzed by flow cytometry. Tumor‐infiltrating NK cells were defined as CD3−CD56⁺ populations. Data were analyzed using FlowJo software. Supernatants were collected after centrifugation (10 000 × g, 10 min, 4 °C), and the concentrations of perforin (liankebio), granzyme B (liankebio), IFN‐*γ* (Invitrogen), and TNF‐*α* (Invitrogen) were quantified using ELISA kits according to the manufacturer's instructions.

### Biosafety Evaluation

At the endpoint of in vivo study (day 28), mice were euthanized and major organs including the liver, kidney, spleen, and heart were harvested, fixed in 4% paraformaldehyde overnight, and embedded in paraffin. Tissue sections (10 µm thick) were stained with hematoxylin and eosin (H&E) following standard protocols. Slides were visualized using a brightfield microscope, and representative images were captured for histological assessment. Whole blood samples were collected from the retro‐orbital sinus of anesthetized mice into EDTA‐coated tubes. Peripheral blood leukocyte counts—including total white blood cells (WBC), neutrophils, lymphocytes, monocytes, eosinophils, and basophils—were measured using an automated hematology analyzer. Data were analyzed to assess systemic immune changes and potential hematotoxicity. Serum samples were obtained by centrifuging whole blood collected in serum‐separation tubes at 3000 × g for 10 min. Biochemical parameters including alanine aminotransferase (ALT), aspartate aminotransferase (AST), alkaline phosphatase (ALP), albumin (ALB), creatinine, and urea levels were determined using an automated biochemical analyzer. These indicators were used to evaluate liver and kidney function and detect potential systemic toxicity induced by treatment.

## Conflict of Interest

The authors declare no conflict of interest.

## Author Contributions

Q.Z., B.Y., and M.S. contributed equally to this work. Q.Z., B.Y., M.S., and J.W. conceptualized the study. B.Y., M.S., Y.Y., M.W., W.Z., Z.L., and J.W. curated the data. Q.Z., B.Y., and M.S. conducted formal analysis. J.W. acquired funding. Q.Z. and B.Y. carried out the investigation. Z.Z. and X.L. developed the methodology. Q.Z., B.Y., M.S., Y.Z., and X.Z. developed the software. Q.Z., B.Y., M.S., J.Z., J.W., K.C., and F.R. wrote the original draft. W.Z., Z.L., and J.W. reviewed and edited the manuscript.

## Supporting information



Supporting Information

## Data Availability

The data that support the findings of this study are available from the corresponding author upon reasonable request.
