# Supplementary material for: Engineered Mesenchymal Stem Cell–NK Cell Complexes for Spatially Targeted and Functionally Revitalized Cancer Immunotherapy
Source: Adv Sci (Weinh). 2025 Aug 19;13(35):e09638. doi: 10.1002/advs.202509638 (PMC13292232; doi:10.1002/advs.202509638)
Supplement: Supplementary file 1 — Supporting Information [file ADVS-13-e09638-s001.docx]

Supporting Information

for

**Engineered Mesenchymal Stem Cell–NK Cell Complexes for Spatially Targeted and Functionally Revitalized Cancer Immunotherapy**

*Qian Zhang^1#^, Bo Yin^1#^, Munaiwaier Sabier^1#^, Youlan Yang^2^, Menglin Wu^2^, Zhouping Zhao^1^, Xuanzhi Luo^1^, Yuehong Zhong^1,5^, Xiuming Zhu^1,6^, Jie Zhang^3^, Jing Wang^3^, Kai Chen^4,7^, Fei Ruan^4,7^, Wei Zhang^3*^, Zhimin Lu^4*^, Jiong Wang^1*^*

1. Department of Geriatric Respiratory and Critical Care, the First Affiliated Hospital of Anhui Medical University, Hefei, 230022, China

2. Department of General Practice, the First Affiliated Hospital of Anhui Medical University, Hefei, 230022, China

3. Department of Obstetrics and Gynecology, the First Affiliated Hospital of Anhui Medical University, Hefei, 230022, China

4. Institute of Medical Genetics and Development, Key Laboratory of Reproductive Genetics (Ministry of Education) and Women’s Hospital, Zhejiang University School of Medicine, Hangzhou, 310006, China

5. Department of Geriatrics, Renhe Hospital of Shanghai University, Shanghai 200431, China

6. Department of Geriatrics Center & National Clinical Research Center for Aging and Medicine, Jing’an District Central Hospital of Shanghai, Fudan University, Shanghai 200040, China

7. Department of Gynecology, Women’s Hospital, Zhejiang University School of Medicine, Hangzhou, 310006, China

# These authors contributed equally.

*Corresponding authors: E-mail: wangjiong@ahmu.edu.cn (J Wang), luzhimin@zju.edu.cn (Z Lu), zhangwei_ahmu@163.com (W Zhang).

**SUPPLEMENTARY FIGURES**

**
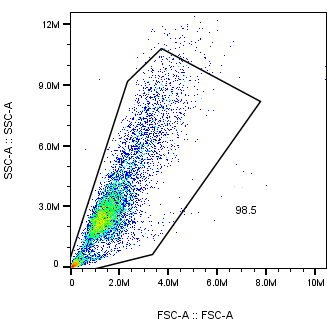
**

**Figure S1. FSC/SSC gating strategy for flow cytometry analysis in Figure 1E.** Forward scatter (FSC) versus side scatter (SSC) plot used to gate all samples analyzed in Figure 1E.

**Figure S2. Time-dependent cytotoxic activity of IL-15-tSC-NK cells against tumor cells.** IL-15-tSC-NK complexes were harvested at different time points (Day 1, 2, 3, 5, and 7) post-manufacturing and co-cultured with LLC tumor cells at for 48 hours. Cytotoxicity was evaluated by LDH release assay. Data are shown as mean ± SD (n = 3); P values were calculated using one-way ANOVA with Tukey’s post-hoc test.

**
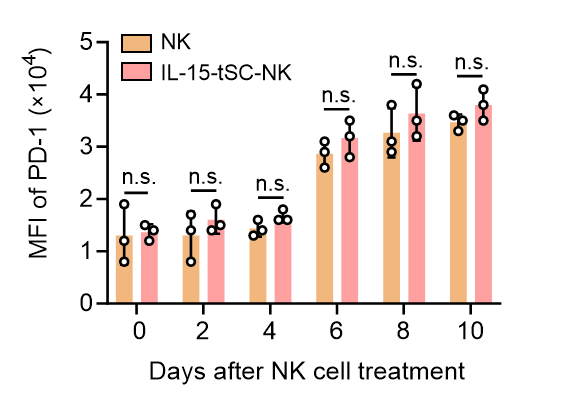
**

**Supplementary Figure S3.** PD-1 expression on NK cells and IL-15-tSC-NK cells over time *in vitro*. Mean fluorescence intensity (MFI) of PD-1 on NK cell was measured by flow cytometry at days 0, 2, 4, 6, 8, and 10 after co-culture. Data are presented as mean ± s.d. (n = 3).


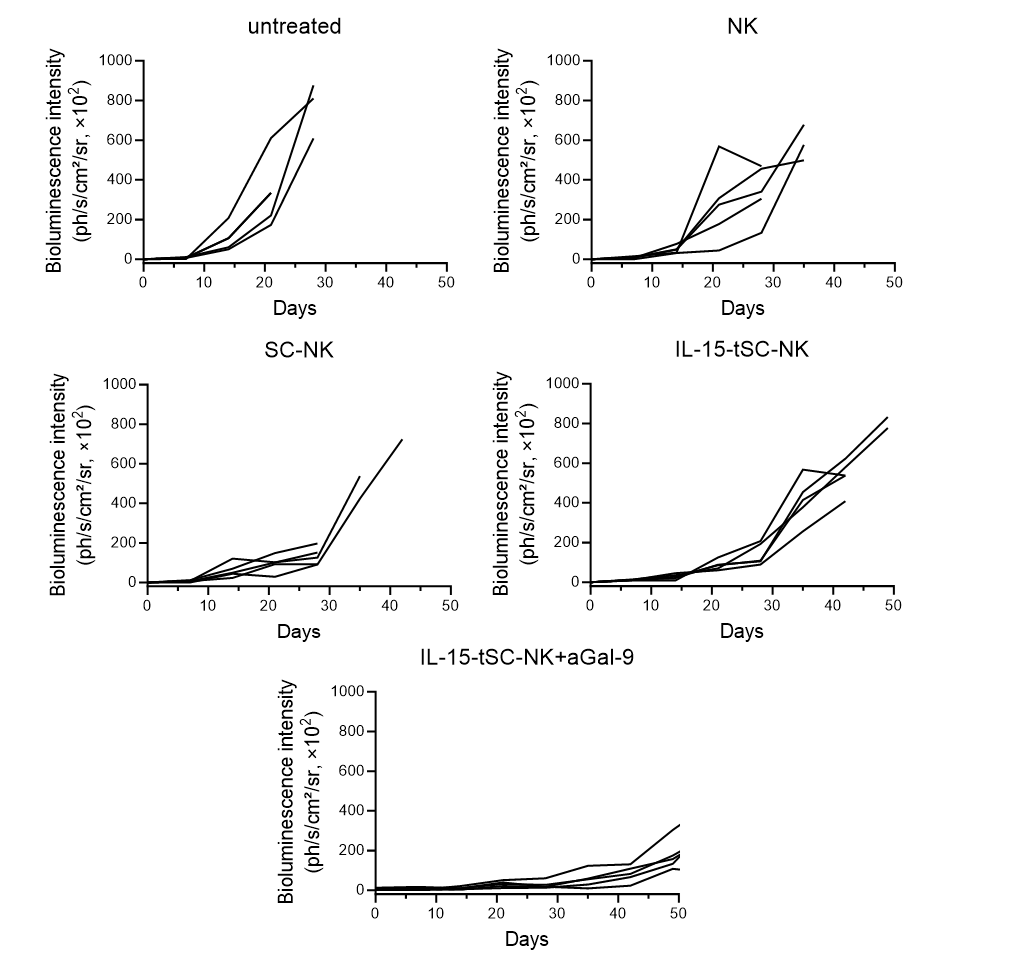


**Supplementary Figure S4.** Quantitative analysis of lung bioluminescence imaging in mice treated with various NK cell-based therapies (n = 5).

**
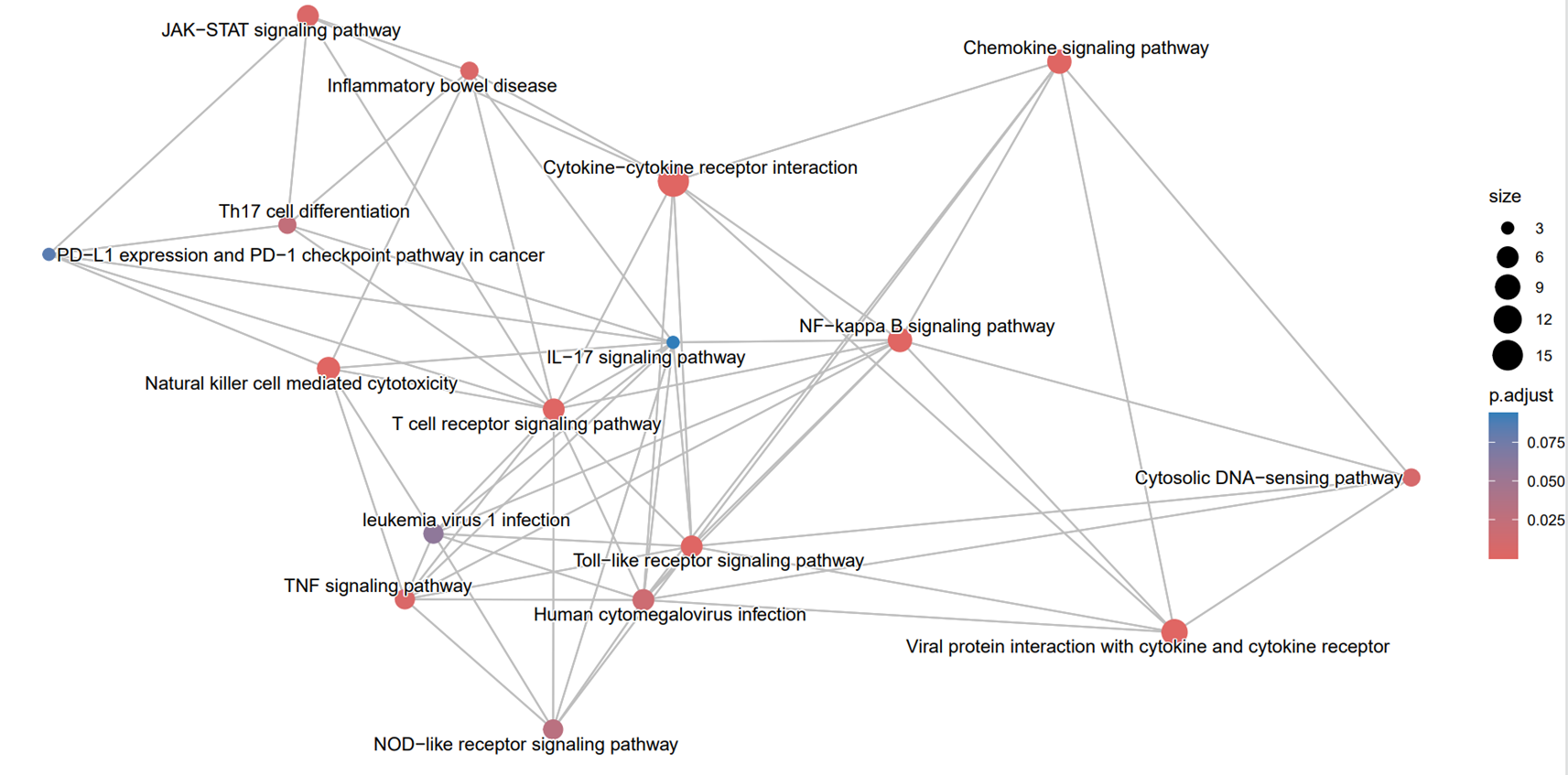
**

**Supplementary Figure S5.** KEGG pathway enrichment network of differentially expressed genes in IL-15-tSC-NK compared to NK cells.


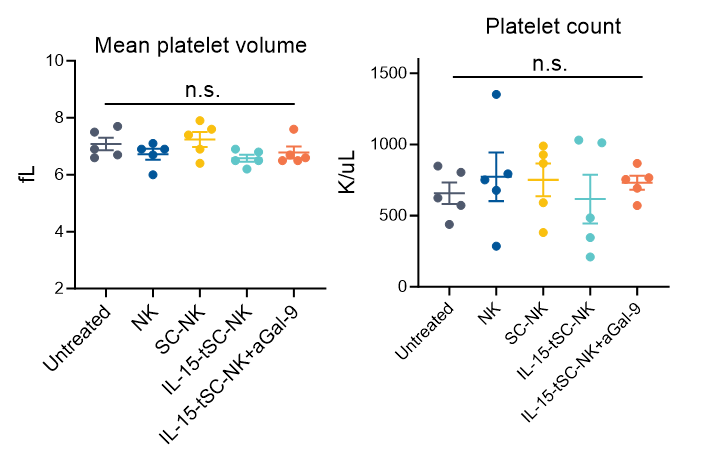


**Supplementary Figure S6.** Mean platelet volume and platelet count in C57 mice received various treatments (n =5).
